# Bariatric Patient Profiles After RYGB and SG Surgery: A 24-Month Observation of Metabolic Changes and Qualitative Malnutrition

**DOI:** 10.3390/nu17172857

**Published:** 2025-09-03

**Authors:** Aleksandra Pankowska, Dariusz Kotlęga, Karina Ryterska, Izabela Gutowska, Maciej Ziętek, Małgorzata Szczuko

**Affiliations:** 1 Independent Provincial Public Hospital Complex in Szczecin-Zdunowo, 70-880 Szczecin, Poland; aleksandra.pankowska@outlook.com; 2 Department of Human Nutrition and Metabolomics, Pomeranian Medical University in Szczecin, 71-460 Szczecin, Poland; karina.ryterska@pum.edu.pl; 3 Department of Pharmacology and Toxicology, University of Zielona Góra, 65-001 Zielona Góra, Poland; 4 Department of Medical Chemistry, Pomeranian Medical University, 70-204 Szczecin, Poland; izabela.gutowska@pum.edu.pl; 5 Department of Perinatology, Obstetrics and Gynecology Pomeranian Medical University in Szczecin, 70-204 Szczecin, Poland; maciej.zietek@pum.edu.pl; 6 Department of Bromatology and Nutritional Diagnostics, Pomeranian Medical University in Szczecin, 71-460 Szczecin, Poland

**Keywords:** bariatric surgery, obesity, Roux-en-Y gastric bypass, sleeve gastrectomy, metabolic parameters, nutritional deficiencies

## Abstract

**Background/Objectives:** Introduction: Bariatric surgery, including laparoscopic sleeve gastrectomy (SG) and Roux-en-Y gastric bypass (RYGB), is an effective treatment for severe obesity and its metabolic complications. This study aimed to assess and compare the clinical outcomes of both procedures over a 24-month follow-up. Few studies have compared 24-month SG and RYGB results in terms of metabolic and nutritional profiles in the Polish cohort. **Materials and Methods:** A retrospective analysis was conducted on 54 patients (27 SG, 27 RYGB) treated between 2018 and 2022. Anthropometric (body weight, BMI), biochemical (lipid profile, glucose, HbA1c, and liver enzymes), and nutritional (iron, ferritin, and vitamin B12) parameters were measured at 1, 6, 12, and 24 months postoperatively. **Results:** Both surgical techniques led to a significant reduction in body weight and BMI during the first postoperative year. After 24 months, weight stabilization was observed in the RYGB group, while statistically significant weight regain occurred in the SG group (*p* < 0.0001). HDL levels significantly increased and triglyceride levels decreased in both groups (*p* < 0.0001), with no significant changes in LDL levels. AST, ALT, decreased dramatically at the first measurement in both methods, while a greater decrease in glycemia was recorded with the SG method (at the same time). A significant reduction in ferritin and vitamin B12 levels was observed in both groups but was more pronounced after RYGB. Iron levels increased until 12 months, followed by a decline by month 24. **Conclusions:** Both RYGB and SG are effective for weight loss and metabolic improvement. RYGB demonstrates greater long-term weight stability but carries a higher risk of nutritional deficiencies. SG should be the first method to consider due to its lower risk, invasiveness, and lower risk of nutritional deficiencies.

## 1. Introduction

Obesity is one of the most serious public health challenges of the 21st century, and its prevalence in the global population is steadily increasing. Currently, obesity affects approximately one-third of the world’s population, and projections indicate that by 2030, as many as 57.8% of adults will be overweight. Obesity is a serious health problem in Poland, affecting approximately 30% of adults, placing the population in the upper half of the European ranking [1]. Obesity is associated with an increased risk of numerous comorbid conditions, including type 2 diabetes, hypertension, dyslipidemia, cardiovascular diseases, degenerative joint diseases, and malignant cancers, as well as sleep disorders and mental health conditions, significantly reducing both the quality and length of life [2]. A more complex definition of obesity was proposed in the latest recommendations, encompassing not only excess adipose tissue but also metabolic and functional criteria [3]. Within this framework, a distinction is made between preclinical obesity (characterized by excess fat tissue without organ dysfunction) and clinical obesity (a chronic systemic disease leading to tissue and organ damage due to disordered adipogenesis and metabolism). This classification also considers additional indicators, such as waist circumference, lipid levels, and other metabolic markers, which may contribute to more effective prevention and treatment of obesity. In response to the escalating obesity epidemic and its associated metabolic complications, bariatric surgery has gained recognition as one of the most effective forms of therapy. The most commonly used surgical methods include sleeve gastrectomy (SG) and Roux-en-Y gastric bypass (RYGB), both of which not only result in sustained weight loss but also improve metabolic parameters and patients’ quality of life [4,5,6]. Bariatric surgery encompasses three primary categories of operative techniques aimed at weight reduction: restrictive procedures (which limit the stomach’s volume), malabsorptive procedures (which reduce the absorptive surface of the gastrointestinal tract by shortening intestinal transit and limiting contact between food content and digestive enzymes), and combined methods that incorporate both mechanisms [7,8]. Sleeve gastrectomy (SG) is primarily a restrictive procedure that involves surgically removing approximately 80% of the stomach’s volume. This reduction limits food intake and also decreases the secretion of ghrelin—the hormone responsible for the sensation of hunger—further enhancing the therapeutic effect [9,10]. Currently, according to data from the International Federation for the Surgery of Obesity and Metabolic Disorders (IFSO), SG is the most frequently performed bariatric procedure worldwide [11]. Roux-en-Y gastric bypass (RYGB) is a combined technique that includes a restrictive component—by creating a small gastric pouch with a volume of 15–20 mL—and a malabsorptive component, achieved by bypassing a significant portion of the stomach and 100–200 cm of the proximal small intestine from the alimentary passage [12,13,14]. This configuration allows for reducing both caloric intake and absorption and also triggers a range of metabolic changes, including modulation of the gut microbiota and improvement of the gut–brain axis function, which contributes to the long-term therapeutic effectiveness [15]. Every surgical intervention carries a risk of adverse effects, which can vary in nature and severity. This also applies to bariatric surgery, where postoperative complications are classified as early—occurring in the immediate postoperative period—and late, which develop over a longer period following the procedure (Table 1) [16,17]. The nutritional status of a patient after bariatric surgery can be significantly impaired not only due to the procedure itself but also because of factors present in the preoperative period. The most important of these include pre-existing nutritional deficiencies, recurrent vomiting, food intolerances, and improper dietary patterns [18,19].

Nutritional deficiencies in individuals with obesity are multifactorial. The main causes include excessive consumption of high-energy, nutrient-poor foods; limited bioavailability of nutrients; chronic inflammation affecting, among other things, iron metabolism; and the presence of small intestinal bacterial overgrowth (SIBO), which disrupts the absorption of micronutrients such as thiamine, vitamin B12, and fat-soluble vitamins. Among the most common deficiencies in patients qualifying for bariatric surgery are deficits of vitamin B12, iron, folic acid, vitamin D, and thiamine [20,21]. Therefore, early dietary intervention and the implementation of vitamin and mineral supplementation in the preoperative period are crucial, especially in individuals with confirmed deficiencies. Postoperatively, supplementation should be continued as a preventive measure, while therapeutic doses are necessary in cases of clinical deficiencies [22,23]. Both sleeve gastrectomy (SG) and Roux-en-Y gastric bypass (RYGB) carry the risk of complications that can impact quality of life and long-term treatment outcomes. Providing interdisciplinary postoperative care and educating patients about potential risks form the foundation of effective bariatric therapy [24,25,26]. There is a lack of data from long-term studies from Central Europe comparing metabolic outcomes and health improvements after various bariatric surgeries. The aim of the study was to evaluate the impact of bariatric surgery using the SG and RYGB methods on selected biochemical and anthropometric parameters, as well as to compare the effectiveness of weight loss over a 24-month observation period.

## 2. Materials and Methods

The study utilized retrospective clinical data obtained from the medical records of patients who underwent surgery and were monitored at the Independent Public Provincial Integrated Hospital in Szczecin-Zdunowo between 2018 and 2022. A total of 54 patients who had undergone surgical treatment for obesity were included in the analysis—27 patients underwent sleeve gastrectomy (SG), and 27 underwent Roux-en-Y gastric bypass (RYGB). The ASMBS guidelines were used in bariatric surgery and clinical practice guidelines for the perioperative nutrition [27,28]

Inclusion criteria:

- Body mass index BMI of 40 kg/m^2^ or higher.
- BMI in the range of 35–40 kg/m^2^ along with the presence of at least one obesity complication. Complications of obesity included type 2 diabetes, hypertension, cardiovascular disease, obesity-induced sleep apnea and/or hypoventilation syndrome, joint disease requiring surgery, non-alcoholic steatohepatitis, non-alcoholic fatty liver disease, hyperlipidemia, female infertility, polycystic ovary syndrome (PCOS), as well as significant social/psychological indications.
- Individuals in whom excessive body weight is a significant disqualifying factor from another form of surgical treatment in the fields of orthopedics, neurosurgery or general surgery for the treatment of hernias, among others.

Exclusion criteria:

- Absolute contraindications to the procedures were patients with incurable diseases leading to cachexia, such as active cancer or acquired immunodeficiency syndrome (AIDS).
- Patients with a short history of life-threatening diseases, such as a recent myocardial infarction or chronic obstructive pulmonary disease (COPD) of a significant degree, and those with endocrine disorders leading to secondary causes of obesity-related disease (Cushing’s syndrome) or severe coagulation disorders.
- People who are uncooperative or do not accept the effects of the treatment (people who are actively addicted to psychoactive substances). People with mental illnesses who, despite treatment and pharmacotherapy, do not achieve the desired therapeutic effect.
- People with severe intellectual disability as well as the period of one year preceding planned pregnancy, as well as pregnancy and lactation.

Only two patients were not included in the study due to missing two consecutive examination dates.

Multi-ingredient supplementation was recommended for all patients after both bariatric surgeries [28,29].

### 2.1. Group Characteristics

In the group of patients who underwent sleeve gastrectomy (SG), women accounted for 88.89% (*n* = 24) and men for 11.11% (*n* = 3). In the group operated on using the Roux-en-Y gastric bypass (RYGB) technique, women made up 85.19% (*n* = 23) and men 14.81% (*n* = 4). The average age of the participants was 42.83 years (SD = 8.98). When divided by surgical method, the mean age of patients who underwent Roux-en-Y gastric bypass was 43.67 years (SD = 9.97), while in the SG group, it was 42.00 years (SD = 7.96). The average height of the entire study population was 167.17 cm (SD = 6.76); the average heights in the RYGB and SG groups were 167.78 cm (SD = 6.95) and 166.56 cm (SD = 6.65), respectively. Preoperative body weight averaged 115.22 kg (SD = 14.86) across the entire group. The mean weight was 114.00 kg (SD = 14.03) in the RYGB group and 116.44 kg (SD = 15.81) in the SG group. The average waist circumference in the total sample was 118.39 cm (SD = 12.19), with 116.67 cm (SD = 13.81) in the RYGB group and 119.52 cm (SD = 11.20) in the SG group. Hip circumference averaged 128.84 cm (SD = 11.01) for the whole group, with values of 127.47 cm (SD = 10.07) in the RYGB group and 129.74 cm (SD = 11.71) in the SG group. The average preoperative body mass index (BMI) was 41.15 kg/m^2^ (SD = 4.08) for the entire study population. This value was 40.46 kg/m^2^ (SD = 4.08) in the RYGB group and 41.84 kg/m^2^ (SD = 4.03) in the SG group. In the SG group, over 85% of patients (*n* = 23) had at least one comorbidity, while only 14.81% (*n* = 4) had no additional health burdens. In the RYGB group, almost all patients suffered from at least one comorbid condition. The most commonly diagnosed diseases included hypertension, type 2 diabetes, gastroesophageal reflux disease (GERD), metabolic disorders—particularly insulin resistance—degenerative joint disease, and hypothyroidism. Less frequently reported diagnoses included dyslipidemia, polycystic ovary syndrome (PCOS), psoriasis, migraines, schizophrenia, depression, and anxiety disorders. The results indicate significant variability in the health status of patients qualified for surgical obesity treatment, with metabolic and endocrine disorders being the most predominant. The biochemical characteristics of the pre-bariatric surgery group are presented below (Table 2).

### 2.2. Biochemical Parameter Assessment

Biochemical parameters were measured based on venous blood samples collected from the patients. Blood was drawn in an outpatient setting, in a seated position, after a fasting period of at least 8 h. Samples were obtained from the antecubital vein in accordance with current guidelines and standard laboratory diagnostic procedures for biological material collection [30]. The biochemical analyses were performed in the accredited diagnostic laboratory affiliated with the hospital. Biochemical measurements were conducted using standard laboratory methods with appropriate analyzers. Total cholesterol, HDL cholesterol, and triglycerides were measured using the enzymatic colorimetric method with the Integra 400 Plus biochemical analyzer (Roche Diagnostics, Warsaw, Poland). LDL cholesterol values were calculated indirectly using the Friedewald formula based on previously obtained direct measurement results. Liver enzyme activity—alanine aminotransferase (ALT) and aspartate aminotransferase (AST)—was determined using the kinetic method in accordance with IFCC standards, and the Integra 400 Plus analyzer was used. Glucose and glycated hemoglobin (HbA1c) concentrations were assessed using the colorimetric method using a diazo reaction on the same device (Integra 400 Plus); the method for HbA1c measurement complied with IFCC guidelines. Iron levels were determined using a colorimetric method, while vitamin B12 and ferritin levels were analyzed using the electrochemiluminescence (ECLIA) technique on the Cobas E411 analyzer (Roche Diagnostics, Warsow, Poland). Standard units for carbohydrates and lipids were used: mg/dL and pg/mL for vitamin B12.

The unit conversion factor should be as follows:

1 mg/dL = 1:18 mmol/L

1 pg/mL = pmol/L × 0.272

The reference range is shown below [31,32,33] (Table 3)

### 2.3. Anthropometric Measurements

To assess the anthropometric parameters of the study participants—such as body weight and height—an electronic scale with an integrated stadiometer (SECA, model M799x1D07-09-032, Hamburg, Germany) was used, offering a measurement accuracy of up to 0.1 kg. Measurements were conducted under standardized conditions—patients were weighed in light clothing, without shoes, in a standing position. To measure waist and hip circumference, a flexible tailor’s tape was used, allowing measurements with an accuracy of up to 0.1 cm. Waist circumference was measured in a standing position, with the patient maintaining a natural posture and without contracting the abdominal muscles. The measurement was taken at the narrowest part of the torso—typically 1–2 cm above the navel, between the lower edge of the rib cage and the iliac crest. The tape was held parallel to the floor and in contact with the skin without compressing it. Hip circumference was also measured in a standing position, with feet together and the body relaxed. The measurement was taken at the widest part of the buttocks, usually located a few centimeters below the iliac crest.

### 2.4. Statistical Analysis

Descriptive statistics were used to present the results for variables measured on nominal and ordinal scales, including frequencies (*n*) and percentages (%). For quantitative variables, the arithmetic mean ($\bar{x}$) and standard deviation (SD) were used. Because the distribution of the studied variables was close to normal, the Shapiro–Wilk test was used. Comparisons between two independent groups were performed using the independent samples Student’s *t*-test. To analyze changes in parameter values at different time points, one-way analysis of variance (ANOVA) was used, supplemented by Tukey’s post hoc test to identify statistically significant differences between specific measurements. In all statistical analyses, a significance level of α = 0.05 was considered the critical threshold. Statistical analyses were performed using Statistica software, version 13.3 (StatSoft, Krakow, Poland).

## 3. Results

Analysis of changes over time (1 month/6 months/12 months/24 months) in the entire study group (women and men), separately for RYGB and SG surgeries (Table 4A,B).

In both groups (RYGB and SG), a significant increase in HDL levels was observed, especially up to the 12th month after surgery (*p* < 0.0001). In the SG group, the HDL increase was greater than in the RYGB group; however, no further significant changes were found between the 12th and 24th months (*p* = 0.104). LDL levels remained stable throughout the observation period in both groups (*p* > 0.27). A significant decrease in triglycerides (TG) was observed in both groups up to the 12th month (*p* < 0.0001), after which the values stabilized. The increase in total cholesterol (TC) was statistically significant only in selected comparisons—in the RYGB group between the 1st and 24th months (*p* = 0.0228), and in the SG group starting from the 6th month (*p* < 0.01) (Table 4A,B).

In both groups (RYGB and SG), a significant reduction in body weight and BMI was observed during the first year after surgery. In the RYGB group, the average body weight decreased from 98.30 kg (1 month) to 74.37 kg (12 months), and BMI from 34.93 to 26.44. After 24 months, a slight increase in body weight and BMI was observed (to 75.81 kg and 26.97, respectively). In the SG group, body weight decreased from 100.70 kg to 75.07 kg (12 months), and BMI from 36.16 to 26.99. After 24 months, a moderate increase in both parameters was recorded (to 77.85 kg and BMI 27.96) (Table 5A,B).

In the RYGB group, a statistically significant decrease in body weight was observed between the 1st and 6th, 12th, and 24th months (*p* < 0.0001). Additionally, a significant change occurred between the 6th and 24th months (*p* = 0.0244), while the change between the 12th and 24th months was not significant (*p* = 0.311). For the BMI, significant changes were observed in all comparisons up to the 12th month (*p* < 0.0001), as well as between the 6th and 24th months (*p* = 0.0322), with no significant change between the 12th and 24th months (*p* = 0.3419). In the SG group, body weight and BMI changed significantly in all analyzed time intervals (*p* < 0.0001). Between the 12th and 24th months, despite statistical significance, an increase in both body weight and BMI was noted (Table 5A,B).

In both groups, a decrease in aminotransferase activity was observed during the follow-up period. In the RYGB group, AST activity decreased from 27.30 U/L (1 month) to 18.70 U/L (24 months), and ALT from 32.72 U/L to 23.12 U/L. In the SG group, AST values dropped from 24.38 U/L to 18.69 U/L, and ALT from 34.93 U/L to 24.48 U/L. The most significant changes were observed up to the 6th month. Fasting glucose and HbA1c levels showed a decreasing trend in both groups, although the changes were moderate. In the RYGB group, glucose decreased from 93.88 mg/dL to 90.38 mg/dL, and HbA1c from 5.23% to 5.18%. In the SG group, glucose dropped from 92.70 mg/dL to 88.33 mg/dL, and HbA1c from 5.31% to 5.28% (Table 6A,B).

In the RYGB group, AST activity showed a significant decrease between the 1st and the 6th, 12th, and 24th months (*p* ≤ 0.0047), while no significant differences were observed at later time points (*p* > 0.92). A similar trend was observed in the SG group, where significant changes occurred during the first year (*p* < 0.015), but not between the 12th and 24th months. For ALT in the RYGB group, a significant decrease was noted between the 1st and the 6th, 12th, and 24th months (*p* ≤ 0.0406), with no significant changes after the 6th month. In the SG group, ALT decreased significantly up to the 12th month (*p* < 0.021), whereas the difference between the 1st and 24th months did not reach significance (*p* = 0.0608). Regarding fasting glucose, a significant change was found only in the SG group between the 1st and 12th months (*p* = 0.0200). No significant changes were observed in the RYGB group (*p* > 0.19). Glycated hemoglobin (HbA1c) remained stable in both groups—no significant differences were found in any of the comparisons (*p* > 0.75) (Table 6A,B)

In both groups, an increase in serum iron (Fe) concentration was observed during the first 12 months after the procedure, followed by a slight decrease at 24 months. The values were higher in the SG group, where the mean Fe concentration rose from 70.00 µg/dL to 95.54 µg/dL (12 months), maintaining a level of 90.92 µg/dL at 24 months. In the RYGB group, the increase was less pronounced—from 64.54 µg/dL to 89.21 µg/dL—followed by a decrease to 73.13 µg/dL. Ferritin showed a downward trend in both groups, more marked in the RYGB group (from 123.48 to 57.12 ng/mL). In the SG group, the values were higher and the decline was milder (from 138.25 to 69.88 ng/mL at 24 months). Vitamin B12 concentration decreased in both groups during the first six months post-surgery and remained at a lower level throughout the observation period. The decline was more pronounced in the RYGB group—from 547.43 pg/mL to 362.80 pg/mL (12 months) and 371.90 pg/mL (24 months). In the SG group, values were somewhat more stable, though also lower after 24 months (340.13 pg/mL) (Table 7A,B).

In both groups, a significant increase in serum iron concentration was observed between the 1st and 12th months (RYGB: *p* = 0.0043; SG: *p* = 0.0064). In the RYGB group, a small but significant decrease was also noted between the 12th and 24th months (*p* = 0.0389), whereas in the SG group, the increase in iron between the 1st and 24th months was also significant (*p* = 0.0389). Ferritin levels significantly decreased in both groups. In the RYGB group, the decrease occurred from the 1st to the 24th month (*p* < 0.005), with additional significant differences between the 6th and 12th and the 6th and 24th months (*p* < 0.013). In the SG group, significant ferritin decreases were observed between the 1st and 6th, 1st and 24th, 6th and 24th, and 12th and 24th months (*p* < 0.05). For vitamin B12, a significant decrease was noted in both groups between the 1st and 6th, 12th, and 24th months (*p* ≤ 0.0022). After the 6th month, the changes were no longer significant (Table 6A,B). Comparison of changes in differences in the studied parameters in the overall study group (women and men) after RYGB and SG procedures. Tables S1–S4 are provided as an appendix in the form of a Supplementary Material.

## 4. Discussion

The aim of this study was to evaluate the profile of patients undergoing bariatric surgery by Roux-en-Y gastric bypass (RYGB) and sleeve gastrectomy (SG) over a 24-month observation period, with particular emphasis on changes in selected anthropometric and biochemical parameters. The results obtained confirm the high effectiveness of both surgical methods for obesity treatment in terms of body weight reduction and improvement of metabolic indices, which are consistent with existing scientific reports.

### 4.1. Lipid Profile Changes (HDL, LDL, TG)

Regardless of the surgical technique applied, a statistically significant increase in HDL cholesterol concentration was observed at successive time points, i.e., from 1 to 24 months postoperatively. Notably, this increase was more pronounced in the group of patients undergoing sleeve gastrectomy (SG). The findings are partly consistent with the observations of Szczuko et al. [34], who reported an initial significant decrease in HDL concentration in the first month after SG surgery, followed by a systematic increase reaching 65.73 mg/dL. In comparison, in this study, the mean HDL concentration after 12 months post-SG was 71.73 mg/dL, and after 24 months, 71.78 mg/dL, indicating a more favorable long-term effect. A similar upward trend was shown in the study by Głuszka et al. [35], where a systematic increase in HDL up to 61.9 mg/dL was noted from the first month to over 12 months of observation. Wojciak et al. [36] also observed a rise in HDL levels—from 37 mg/dL in the first month after surgery to 55 mg/dL after 12 months following SG. In the present study, a statistically significant, systematic decrease in triglyceride (TG) levels was found from 1 to 24 months postoperatively. After 12 months following SG, the mean TG concentration was 79.44 mg/dL, confirming the effectiveness of this method in improving the lipid profile. Comparable results were reported by Głuszka et al. [35], with TG values of 111.6 mg/dL after one year, and by Wojciak et al. [36], where the mean LDL cholesterol concentration after 12 months was 113 mg/dL. Szczuko et al. [34] also demonstrated a decrease in TG to 84.32 mg/dL after 12 months post-SG.

For patients operated on by Roux-en-Y gastric bypass (RYGB), the study by Szczuko et al. [34] showed statistically significant decreases in total cholesterol (TC), LDL, and TG. In the present study, statistical significance was confirmed only for the decrease in TG over time after RYGB. No statistically significant differences were found for LDL, while a significant increase in total cholesterol was observed between the 1st and 24th months of follow-up. A similar upward trend in TC was also noted in patients after SG. In the study by Wojciak et al. [36], following SG, a systematic decrease in total cholesterol was observed in the first three months, after which an increase and stabilization occurred from the 6th month onward. A similar pattern was seen for the LDL fraction—initial significant reduction followed by an increase to 122 mg/dL in the 3rd and 6th months postoperatively. In the present study, the mean total cholesterol level after 6 months was 185.52 mg/dL, and LDL was 111.71 mg/dL. The favorable changes in the lipid profile may partly result from the use of hypolipidemic pharmacotherapy in selected patients. Regarding the increase in HDL concentration, an important role may have been played by adequate patient preparation before surgery, including the introduction of moderate physical activity and its continuation after surgery. It is also important to emphasize the role of proper perioperative care, including nutritional education, conducted by an interdisciplinary team. These play a key role in maintaining a stable lipid profile and preventing fatty liver disease in the long-term postoperative observation.

### 4.2. Liver Enzyme and Glucose Metabolism Outcomes

In the study conducted by Szczuko et al. [34], a statistically significant increase in liver enzyme activities (ALT, AST) was observed during the first month after bariatric surgery by the RYGB method, followed by a marked decrease up to the 6th month. A similar trend was demonstrated in the present study regarding alanine aminotransferase (ALT) activity, whereas for aspartate aminotransferase (AST), a statistically significant decrease was observed between the 1st and the 6th, 12th, and 24th months of postoperative observation. These findings are also supported by data presented by Wojciak et al. [36], who reported a systematic and statistically significant reduction in ALT and AST activity from the 1st to the 12th month after surgery. One year after the operation, ALT and AST values were 22 IU/l and 21.05 IU/l, respectively. In comparison, the present study recorded comparable values at 12 months: ALT—22.85 IU/l and AST—17.19 IU/l, indicating a similar pace and direction of changes after following surgical obesity treatment. However, these changes were within the reference range, so it is difficult to speak of a significant benefit. They were within the normal range.

In the study by Szczuko et al. [34], a linear decrease in fasting glucose levels was observed in patients undergoing bariatric surgery by RYGB and SG methods, reaching values of 83.62 mg/dL and 88.12 mg/dL, respectively. A similar trend was observed in the present study—glucose levels at 24 months post-surgery were 90.38 mg/dL in the RYGB group and 88.33 mg/dL in the SG group. Although these changes did not reach statistical significance, they may suggest a positive impact of the surgical procedures on patients’ dietary habits and carbohydrate metabolism regulation. In the study by Wojciak et al. [36], a systematic decrease in fasting glucose was also observed—from 109 mg/dL at one month to 98 mg/dL at 12 months after surgery. A similar trend was noted for glycated hemoglobin (HbA1c), which was 5.3% at one year. In the present analysis, an almost identical value of 5.27% at 12 months was obtained, suggesting effective halting of type 2 diabetes progression. These changes were within the reference range, which limits their usefulness in assessing metabolic improvement or diabetes remission.

### 4.3. Weight Trajectory and Long-Term Stability

Consistent conclusions were also presented by Kaska et al. [37], who studied the dynamics of type 2 diabetes remission after RYGB surgery, analyzing its dependence on baseline BMI. They showed that decreases in key glycemic parameters between 3 and 12 months post-surgery correlated with BMI reduction, although in some cases metabolic improvements occurred earlier, independently of weight loss. A medium-term (3-year) follow-up of patients after RYGB confirmed sustained remission of type 2 diabetes regardless of baseline BMI, underscoring the high efficacy of this surgical procedure in treating this metabolic disease. In the present study, a statistically significant decrease in body weight and BMI was observed at successive time points up to 12 months after bariatric surgery, both by sleeve gastrectomy (SG) and Roux-en-Y gastric bypass (RYGB). These results align with observations by Głuszek et al. [35] and Wojciak et al. [36], who also reported significant BMI reduction within one year after surgery—with mean BMI 12 months after SG of 30.37 in their studies, compared to 26.99 in the present study. Comparative analysis of both surgical procedures revealed no statistically significant differences in body weight and BMI reduction during the first 12 postoperative months, indicating a similar pace of decrease in these parameters regardless of the surgical technique used. Similar conclusions were drawn by Szczuko et al. [34] and Głuszek et al. [35] in their 12-month observations. However, it is worth noting that between the 12th and 24th months after SG, a statistically significant increase in body weight was observed, which may indicate a tendency toward partial weight regain in this patient group. This phenomenon was not observed in the RYGB group, where body weight remained stable both after one and two years post-surgery. Similar differences in the long-term efficacy of both methods were confirmed by Garg et al. [38]. Consistent results were presented by Saeed et al. [39], who confirmed greater efficacy of RYGB in long-term weight control—after 5 years, mean percent weight loss was 74.8% for RYGB compared to 65.1% for SG. Our results do not confirm a significant difference in weight loss between the two methods at 24 months. Poorer adherence to lifestyle recommendations, hormonal differences and patient age may have contributed to this.

### 4.4. Nutritional Deficiencies (Iron, Ferritin, B12)

One of the important issues addressed in this study is the assessment of the frequency of micronutrient deficiencies following bariatric surgery. This problem is widely described in the literature, and its clinical significance emphasizes the need for regular follow-up visits, an interdisciplinary therapeutic approach, and appropriately tailored supplementation in the population of patients undergoing surgical treatment for obesity [40,41]. In the present study, a statistically significant decrease in vitamin B12 (cobalamin) levels was observed at successive time points—between the 1st, 6th, 12th, and 24th months of follow-up—both in patients after RYGB and SG. Importantly, no significant differences were found between the surgical groups at any analyzed time point, which may indicate a similar susceptibility of both procedures to the development of cobalamin deficiencies. Similar conclusions were presented by Silva et al. [42], who also confirmed a tendency for decreased vitamin B12 levels after bariatric surgery, noting that patients undergoing RYGB were significantly more at risk of developing deficiencies compared to those subjected to other types of surgical procedures. The development of micronutrient deficiencies after bariatric surgery is influenced not only by anatomical factors, such as malabsorption due to altered gastrointestinal tract anatomy or reduced volume of consumed meals, but also by behavioral factors. Food selectivity is particularly common, manifested by an aversion to foods rich in vitamin B12—primarily meat, and to a lesser extent fish and eggs. If we define values in the range of 200–400 pg/mL (200–400 ng/l) as borderline, it turns out that almost all patients after both RYGB and SB had cobalamin deficiencies after a period of 12 months. These factors should be considered in the planning of long-term care and nutritional education for patients after bariatric surgery. Serum vitamin B12, concentrations indicate that if the lower limit of normal (200 ng/L or 147 pmol/L) is used, patients with elevated methylmalonic acid (MMA) levels will be missed [33]. Concerns have arisen regarding the use of serum vitamin B12 measurements alone because the interpretation of intermediate range vitamin B12 concentrations is unclear [33]. MMA is considered a specific marker of cobalamin metabolism, and elevated tHcy is seen in vitamin B12 deficiency, along with folate and vitamin B6 deficiencies. Plasma tHcy concentrations are elevated in cases of renal failure or polymorphisms in methylenetetrahydrofolate reductase (MTHFR), which should be considered in bariatric patient care.

Analysis of ferritin levels in patients after bariatric surgery revealed a statistically significant decrease at successive time points in the group operated on with the Roux-en-Y gastric bypass (RYGB) method. In patients after sleeve gastrectomy (SG), a statistically significant decrease in ferritin was observed between the 1st and 6th months and between the 1st and 24th months of observation. Nevertheless, nearly 20% of patients had levels reduced below 20 ug/mL). When comparing both groups, no statistically significant differences in ferritin levels were noted in most analyzed periods. The exception was the period between the 12th and 24th months postoperatively, during which a significantly greater decrease in ferritin concentration was observed in the SG group compared to the RYGB group. This may suggest greater susceptibility of patients after SG to developing iron deficiency over the longer term, highlighting the need for individualized monitoring of iron metabolism parameters and implementation of appropriate supplementation depending on the surgical technique used. In patients undergoing Roux-en-Y gastric bypass (RYGB), a statistically significant increase in serum iron concentration was observed between the 1st and 12th months postoperatively. Similarly, in the sleeve gastrectomy (SG) group, a significant increase in iron levels occurred between the 1st and 24th months of follow-up. No significant differences were found between the surgical groups in any analyzed period regarding changes in iron concentration.

These results may suggest the effectiveness of implemented recommendations concerning regular follow-up visits and daily supplementation with iron-containing preparations, starting from the perioperative period through to the end of the 24-month observation. Different conclusions were drawn by Silva et al. [39], who in their analysis reported widespread micronutrient deficiencies, particularly in patients after RYGB surgery. The most common deficiency was iron (21.3%), followed by vitamin B12 (16.9%) and, to a lesser extent, folic acid (4.5%). For these parameters, the reference range is wide, so the authors tried to avoid interpreting the range and focused on the nearly 40% loss of ferritin and cobalamin after surgery in the long term. These findings emphasize the need for strict monitoring of nutritional status and comprehensive care of patients after bariatric procedures, including both dietary and pharmacological aspects [41,42,43]. It should be noted, however, that a limitation of the present study was the lack of detailed data regarding levels of other mineral components such as zinc, selenium, calcium, and magnesium. Although their metabolism is considered more stable than that of vitamins, the available literature indicates the possibility of clinically significant deficiencies of these elements in the bariatric population [44,45,46], which represents an important area for further research and monitoring.

## 5. Limitations

Limitations of this study include the following:

- Relatively small sample size (*n* = 54) and single-center design;
- Retrospective nature with potential data recording errors;
- Lack of monitoring of dietary/supplementation adherence;
- Lack of information on quality of life or patient-reported outcomes.

## 6. Conclusions

Both procedures (RYGB and SG) proved effective in treating morbid obesity, leading to significant weight loss and improvement in anthropometric and metabolic parameters during the study period. Women constitute the vast majority of patients undergoing both types of bariatric surgery. Long-term analysis (12–24 months) indicates greater stability of results in the RYGB group. Both surgical techniques demonstrated a beneficial effect on carbohydrate and lipid metabolism, which equally confirms their metabolic effectiveness.

Patients after RYGB are more prone to developing nutritional deficiencies in iron, ferritin, and vitamin B12 levels. Measuring only these parameters without homocysteine and methylmalonic acid may be insufficient. Lifelong supplementation and regular laboratory monitoring are necessary to prevent deficiencies and related complications. RYGB may be the preferred procedure for patients with severe metabolic disorders (type 2 diabetes, dyslipidemia) and those at high risk of gastroesophageal reflux disease (GERD), due to its potentially stronger metabolic effect. However, higher treatment costs, increased risk of complications, and greater postoperative care demands should be considered.

Sleeve gastrectomy may be a more suitable option for patients with lower metabolic risk, those contraindicated for RYGB, or with concerns about dumping syndrome and severe nutritional deficiencies. This method may also be preferred in women planning pregnancy, due to a lower risk of nutrient deficiencies that could affect pregnancy course and fetal health. The selection of the appropriate surgical technique should be an individualized process based on a detailed assessment of the patient’s clinical condition, their preferences, and an analysis of the potential benefits and risks associated with the chosen procedure. An interdisciplinary approach and long-term specialist care are key factors determining the success of bariatric treatment.

## Figures and Tables

**Table 1:** Early and late complications after bariatric surgery: SG and RYGB.

Type of Complication	Time of Occurrence	Clinical Characteristics	More Frequent Method
Perioperative bleeding	Early (0–7 days)	Bleeding from the staple line or intraoperative vessels	RYGB, SG
Anastomotic leakage	Early (0–7 days)	Fever, tachycardia, abdominal pain, signs of peritonitis	SG
Gastrointestinal strictures	Early (<30 days)	Difficulty swallowing, vomiting	RYGB, less commonly SG
Thrombosis/embolism	Early (<30 days)	Pulmonary embolism, deep vein thrombosis	RYGB, SG
Gallbladder stones (cholelithiasis)	Late (>1 month)	Biliary colic, cholecystitis	RYGB, SG
Marginal ulcers	Late (>1 month)	Pain, gastrointestinal bleeding	Mainly RYGB
Small bowel obstruction	Late (>1 month)	Abdominal pain, vomiting, absence of peristalsis	RYGB
Internal hernias	Late (>1 month)	Episodic abdominal pain, symptoms of intestinal obstruction	RYGB
Gastroesophageal reflux	Late (>1 month)	Heartburn, acid reflux, esophagitis	SG
Dumping syndrome (DS)	Late (1–3 h after a meal)	Nausea, abdominal pain, sweating, hypoglycemia (early/late dumping syndrome)	Mainly RYGB
Nutritional deficiencies	Late (months to years)	Iron, vitamin B12, folic acid, calcium, and ferritin deficiencies; general and neurological symptoms	RYGB > SG

SG—sleeve gastrectomy; RYGB—Roux-en-Y gastric bypass [16,17].

**Table 2:** The biochemical characteristics of the pre-bariatric surgery patients.

Parameter	RYGB $\bar{x}$ ± SD	SG $\bar{x}$ ± SD	*p*-Value
ALT	28.44 ± 7.29	27.15 ± 15.7	*p* = 0.112
AST	28.17 ± 6.8	28.25 ± 9.71	*p* = 0.451
TG	160.85 ± 91.71	158.04 ± 53.36	*p* = 0.721
TC	203.83 ± 29.22	235.7 ± 33.05	*p* = 0.041
HDL	51.13 ± 12.51	52.44 ± 12.07	*p* = 0.773
LDL	125.37 ± 37.43	137.68 ± 34.65	*p* = 0.373
GLU	105.88 ± 11.18	111.45 ± 16.47	*p* = 0.445
HbA1c %	5.63 ± 0.84	5.82 ± 0.57	*p* = 0.245
CRP	0.49 ± 0.25	0.59 ± 0.31	*p* = 0.036

$\bar{x}$—arithmetic mean; SD—standard deviation; SG—sleeve gastrectomy; RYGB—Roux-en-Y gastric bypass; HDL—High-Density Lipoprotein; LDL—Low-Density Lipoprotein; TG—triglycerides; TC—total cholesterol; AST—aspartate aminotransferase; ALT—alanine aminotransferase; GLU—glucose; HbA1c (%)—glycated hemoglobin.

**Table 3:** Table Summary of Clinical Thresholds.

Parameter	Parameter Normal (Adult)	Deficiency Limit
Vitamin B12	200–800 pg/mL	<200 pg/mL
Ferritin	M: 40–300 ng/mL; W: 20–200 ng/mL	<30 ng/mL (deficient); <15 ng/mL (WHO)
Iron	M: 65–175 µg/dL; W: 50–170 µg/dL	<65 µg/dL M; <50 µg/dL W

M—man; W—women.

**Table 4:** (**A**) Changes in HDL, LDL, TG, and total cholesterol levels after bariatric surgeries (RYGB and SG) at subsequent time points in the overall group (women and men). (**B**) Statistical significance of changes in HDL, LDL, TG, and total cholesterol (TC) concentrations in post hoc analysis in the overall group (women and men) depending on RYGB and SG surgeries at different time intervals.

(**A**)						
**PARAMETER**			**RYGB** $\bar{x}$	**RYGB SD**	**SG** $\bar{x}$	**SG SD**
HDL [1 month]			39.22	6.00	42.50	9.78
HDL [6 months]			49.32	7.13	58.78	12.62
HDL [12 months]			58.93	9.16	67.70	10.88
HDL [24 months]			63.13	10.40	71.78	12.68
LDL [1 month]			89.20	28.22	103.04	33.05
LDL [6 months]			88.01	29.89	111.71	27.21
LDL [12 months]			85.77	25.13	105.85	33.56
LDL [24 months]			88.92	28.00	107.53	39.01
TG [1 month]			110.78	33.00	115.63	30.60
TG [6 months]			89.41	30.23	85.07	22.11
TG [12 months]			77.81	32.60	79.44	31.07
TG [24 months]			77.37	36.10	74.48	29.34
TC [1 month]			145.11	31.98	164.52	34.13
TC [6 months]			152.19	32.73	185.52	34.45
TC [12 months]			157.15	29.08	186.19	36.35
TC [24 months]			164.27	32.74	193.01	41.94
(**B**)						
	**RYGB**					
	1 m vs. 6 m	1 m vs. 12 m	1 m vs. 24 m	6 m vs. 12 m	6 m vs. 24 m	12 m vs. 24 m
HDL	<0.0001	<0.0001	<0.0001	<0.0001	<0.0001	0.0149
LDL	0.9897	0.905	>0.9999	0.9714	0.9986	0.6738
TG	0.0133	0.0003	<0.0001	0.0376	0.1737	0.9996
TC	0.3669	0.1288	0.0228	0.8077	0.2879	0.206
	**SG**					
HDL	<0.0001	<0.0001	<0.0001	<0.0001	<0.0001	0.104
LDL	0.2729	0.9731	0.9419	0.4577	0.8734	0.9559
TG	<0.0001	<0.0001	<0.0001	0.5619	0.0775	0.7074
TC	0.0022	0.0109	0.0087	0.9987	0.5791	0.3318

Lipid concentration units are given in mg/dL; $\bar{x}$—arithmetic mean; SD—standard deviation; SG—sleeve gastrectomy; RYGB—Roux-en-Y gastric bypass; HDL—High-Density Lipoprotein; LDL—Low-Density Lipoprotein; TG—triglycerides; TC—total cholesterol; *p*—statistical significance level; <0.0001—*p*-value less than 0.0001 (highly statistically significant change); 1 m, 6 m, 12 m, 24 m—months after surgery; vs.—versus (compared to); SG—sleeve gastrectomy

**Table 5:** (**A**) Changes in body weight and BMI at successive time points after RYGB and SG surgeries in the overall group (women and men). (**B**) Statistical significance of changes in body weight and BMI in post hoc analysis in the overall group (women and men) after RYGB and SG surgeries (comparisons between consecutive time points).

(**A**)						
**PARAMETER**			**RYGB** $\bar{x}$	**RYGB SD**	**SG** $\bar{x}$	**SG SD**
BW [1 month]			98.30	10.85	100.70	14.70
BW [6 months]			80.59	10.62	82.30	11.64
BW [12 months]			74.37	9.83	75.07	11.98
BW [24 months]			75.81	11.51	77.85	12.90
BMI [1 month]			34.93	3.30	36.16	3.62
BMI [6 months]			28.64	3.60	29.59	3.03
BMI [12 months]			26.44	3.41	26.99	3.25
BMI [24 months]			26.97	4.20	27.96	3.57
(**B**)						
	**RYGB**					
	1 m vs. 6 m	1 m vs. 12 m	1 m vs. 24 m	6 m vs. 12 m	6 m vs. 24 m	12 m vs. 24 m
Body weight	<0.0001	<0.0001	<0.0001	<0.0001	0.0244	0.311
BMI	<0.0001	<0.0001	<0.0001	<0.0001	0.0322	0.3419
	**SG**					
Body weight	<0.0001	<0.0001	<0.0001	<0.0001	0.0071	0.0001
BMI	<0.0001	<0.0001	<0.0001	<0.0001	0.0046	0.0001

Body weight units are given in kilograms; $\bar{x}$—arithmetic mean; SD—standard deviation; SG—sleeve gastrectomy; RYGB—Roux-en-Y gastric bypass; BW—body weight; BMI—body mass index. *p*—level of statistical significance; <0.0001—*p*-value less than 0.0001 (highly statistically significant change); 1 m, 6 m, 12 m, 24 m—months after surgery; vs.—versus (compared to).

**Table 6:** (**A**) Changes in biochemical parameters (AST, ALT, glucose, glycated hemoglobin) in the overall group (women and men) at subsequent time points after RYGB and SG surgeries. (**B**) Statistical significance of changes in AST, ALT, glucose, and glycated hemoglobin (HbA1c) in post hoc analysis in the overall group (women and men) after RYGB and SG surgeries (comparisons between consecutive time points).

(**A**)						
**PARAMETER**			**RYGB** $\bar{x}$	**RYGB SD**	**SG** $\bar{x}$	**SG SD**
AST [1 month]			27.30	10.10	24.38	8.42
AST [6 months]			20.00	8.70	17.38	4.67
AST [12 months]			19.52	5.61	17.19	3.31
AST [24 months]			18.70	7.28	18.69	5.30
ALT [1 month]			32.72	12.64	34.93	19.24
ALT [6 months]			19.60	8.36	21.33	11.86
ALT [12 months]			21.32	8.58	22.85	6.90
ALT [24 months]			23.12	11.12	24.48	9.69
GLU [1 month]			93.88	11.33	92.70	9.57
GLU [6 months]			92.04	9.16	89.33	7.90
GLU [12 months]			91.42	11.32	87.26	9.68
GLU [24 months]			90.38	9.37	88.33	8.49
HbA1c (%) [1 month]			5.23	0.41	5.31	0.28
HbA1c (%) [6 months]			5.23	0.21	5.29	0.27
HbA1c (%) [12 months]			5.20	0.26	5.27	0.33
HbA1c (%) [24 months]			5.18	0.36	5.28	0.25
(**B**)						
	**RYGB**					
	1 m vs. 6 m	1 m vs. 12 m	1 m vs. 24 m	6 m vs. 12 m	6 m vs. 24 m	12 m vs. 24 m
AST	0.0046	0.0006	0.0047	0.9794	0.925	0.9508
ALT	0.0006	0.0047	0.0406	0.7407	0.4701	0.7758
GLU	0.8254	0.7723	0.1986	0.9866	0.8516	0.9396
HbA1c	0.9998	0.985	0.8824	0.9259	0.8233	0.9755
	**SG**					
AST	0.0041	0.0015	0.0147	0.9971	0.7408	0.2217
ALT	0.0187	0.0202	0.0608	0.9158	0.7289	0.7826
GLU	0.2659	0.02	0.0514	0.5487	0.9117	0.9073
HbA1c	0.9393	0.8381	0.7556	0.9672	0.96	0.9993

Units for AST and ALT are given in U/L, glucose in mg/dL, and glycated hemoglobin in %. $\bar{x}$—arithmetic mean; SD—standard deviation; SG—sleeve gastrectomy; RYGB—Roux-en-Y gastric bypass; AST—aspartate aminotransferase; ALT—alanine aminotransferase; GLU—glucose; HbA1c (%)—glycated hemoglobin. *p*—level of statistical significance; <0.0001—*p*-value less than 0.0001 (highly statistically significant change); 1 m, 6 m, 12 m, 24 m—months after surgery; vs.—versus (compared to).

**Table 7:** (**A**) Changes in serum iron, ferritin, and vitamin B12 concentrations in the overall group (women and men) after bariatric surgeries (RYGB and SG). (**B**) Statistical significance of changes in serum iron, ferritin, and vitamin B12 concentrations in post hoc analysis in the overall group (women and men) after RYGB and SG (comparisons between consecutive time points).

(**A**)						
**PARAMETER**			**RYGB** $\bar{x}$	**RYGB SD**	**SG** $\bar{x}$	**SG SD**
Fe [1 month]			64.54	17.83	70.00	26.39
Fe [6 months]			79.58	27.60	85.00	38.18
Fe [12 months]			89.21	28.19	95.54	40.05
Fe [24 months]			73.13	33.96	90.92	37.34
ferritin [1 month]			123.48	139.40	138.25	156.38
ferritin [6 months]			84.56	87.56	100.55	107.90
ferritin [12 months]			60.21	64.68	91.49	89.16
ferritin [24 months]			57.12	74.26	69.88	73.84
B12 vitamin [1 month]			547.43	202.90	551.77	220.77
B12 vitamin [6 months]			398.77	147.70	376.80	103.14
B12 vitamin [12 months]			362.80	148.18	375.79	87.02
B12 vitamin [24 months]			371.90	190.12	340.13	87.96
(**B**)						
	**RYGB**					
	1 m vs. 6 m	1 m vs. 12 m	1 m vs. 24 m	6 m vs. 12 m	6 m vs. 24 m	12 m vs. 24 m
Fe	0.0527	0.0043	0.645	0.4526	0.7531	0.0389
ferritin	0.0275	0.005	0.0037	0.0084	0.0125	0.9571
B12 vitamin	0.0022	<0.0001	0.0009	0.39	0.8465	0.9856
	**SG**					
Fe	0.0829	0.0064	0.0389	0.4489	0.7543	0.934
ferritin	0.0146	0.0545	0.0103	0.6413	0.0462	0.0187
B12 vitamin	0.0003	0.0006	<0.0001	0.9999	0.1355	0.1513

Iron and ferritin concentrations are given in µg/L—micrograms per liter; vitamin B12 is given in pg/mL—picograms per milliliter; $\bar{x}$—arithmetic mean; SD—standard deviation; Fe—iron; SG—sleeve gastrectomy; RYGB—Roux-en-Y gastric bypass. *p*—level of statistical significance; <0.0001—*p*-value less than 0.0001 (highly statistically significant change); vs.—versus (compared to).

## Data Availability

The data presented in this study are available on request from the first and corresponding author.

## Supplementary Materials

The following supporting information can be downloaded at: https://www.mdpi.com/article/10.3390/nu17172857/s1, Table S1. Comparison of changes in lipid parameter differences in the total group (women and men) depending on the type of surgery (RYGB and SG) at various time points. Table S2. Comparison of changes in the difference in vitamin B12, ferritin, and iron (Fe) levels in the overall group (women and men) depending on the type of surgery (RYGB and SG) at different time intervals. Table S3. Comparison of changes in body weight and BMI differences in the overall group (women and men) depending on the type of surgery—RYGB and SG—at different time intervals. Table S4. Comparison of changes in biochemical parameters (AST, ALT, glucose, glycated hemoglobin) in the overall group (women and men) depending on the type of surgery (RYGB vs. SG) at different time intervals.

## Abbreviations

The following abbreviations are used in this manuscript:

ALT	alanine aminotransferase
AST	aspartate aminotransferase
BMI	body mass index
BW	body weight
GLU	glucose
HbA1c	glycated hemoglobin
RYGB	Roux-en-Y gastric bypass
SG	Sleeve gastrectomy
