# Supplementary material for: Bariatric Patient Profiles After RYGB and SG Surgery: A 24-Month Observation of Metabolic Changes and Qualitative Malnutrition
Source: Nutrients. 2025 Sep 3;17(17):2857. doi: 10.3390/nu17172857 (PMC12430445; doi:10.3390/nu17172857)
Supplement: Supplementary file 1 [file nutrients-17-02857-s001.zip › nutrients-3839182-supplementary.pdf]

SUPPLEMENT

Table S1. Comparison of changes in lipid parameter differences in the total group (women and men) depending on the type of surgery (RYGB and SG) at various time points

PARAMETER	DIFFERENCE	RYGB x̄	RYGB SD	SG x̄	SG SD	p
HDL	[6m-1m]	10.10	6.62	16.28	9.77	0.0089
	[12m-1m]	19.71	8.40	25.20	9.12	0,.0253
	[24m-1m]	23.90	10.33	29.28	11.44	0.0757
	[12m-6m]	9.60	8.63	8.92	8.51	0.7725
	[24m-6m]	13.80	10.03	13.00	11.69	0.7887
	[24m-12m]	4.20	6.66	4.08	8.87	0.9551
LDL	[6m-1m]	-1.19	20.10	8.67	24.33	0.1105
	[12m-1m]	-3.43	26.35	2.81	34.09	0.4550
	[24m-1m]	-0.28	30.84	4.49	41.43	0.6330
	[12m-6m]	-2.24	26.59	-5.86	20.44	0.5772
	[24m-6m]	0.91	30.47	-4.18	28.73	0.5306
	[24m-12m]	3.15	14.46	1.68	17.14	0.7353
TG	[6m-1m]	-21.37	33.42	-30.56	21.95	0.2380
	[12m-1m]	-32.96	35.57	-36.19	26.85	0.7087
	[24m-1m]	-33.41	31.86	-41.15	27.69	0.3451
	[12m-6m]	-11.59	20.96	-5.63	22.24	0.3154
	[24m-6m]	-12.04	29.52	-10.59	21.70	0.8385
	[24m-12m]	-0.44	22.84	-4,96	23.99	0.4816
TC	[6m-1m]	7.07	22.21	21.00	26.96	0.0433
	[12m-1m]	12.04	27.43	21.67	33.06	0.2494
	[24m-1m]	19.16	32.19	28.50	42.31	0.3657
	[12m-6m]	4.96	28.83	0.67	22.72	0.5457
	[24m-6m]	12.09	34.54	7.50	30.26	0.6058

[Xmo–Ymo] – change in the parameter value between the time point X months post-operation and the time point Y months post-operation (e.g., [6m–1m] indicates the change between the 6th and 1st month); x̄ – arithmetic mean; SD – standard deviation; p – level of statistical significance; SG – Sleeve Gastrectomy; RYGB – Roux-en-Y Gastric Bypass; HDL – High-Density Lipoprotein; LDL – Low-Density Lipoprotein; TG – triglycerides; TC – total cholesterol.

Comparative analysis showed that the increase in HDL concentration was significantly greater in the SG group between the 1st and 6th month (p = 0.0089) and between the 1st and 12th month after surgery (p = 0.0253). No significant differences between the groups were observed in the subsequent time intervals. For LDL, triglycerides (TG), and total cholesterol (TOTAL CHOL.), no significant differences between the groups were found in most observation periods (p > 0.05). An exception was the increase in TOTAL CHOL. between the 1st and 6th month, which was significantly greater in the SG group (p = 0.0433).

Table S2. Comparison of changes in the difference of vitamin B12, ferritin, and iron (Fe) levels in the overall group (women and men) depending on the type of surgery (RYGB and SG) at different time intervals

**PARAMETER**	**DIFFERENCE**	**RYGB x̄**	**RYGB SD**	**SG x̄**	**SG SD**	**p**
B12 vitamin	[6m-1m]	-148.66	190.67	-174.97	190.58	0.6143
	[12m-1m]	-184.63	180.28	-175.98	200.53	0.8683
	[24m-1m]	-175.52	207.06	-211.63	202.95	0.5204
	[12m-6m]	-35.97	116.00	-1.01	79.89	0.2029
	[24m-6m]	-26.86	171.14	-36.67	84.53	0.7906
	[24m-12m]	9.11	137.32	-35.65	84.41	0.1551
Ferritin	[6m-1m]	-38.92	67.14	-37.70	59.69	0.9444
	[12m-1m]	-63.26	88.37	-46.76	89.89	0.4993
	[24m-1m]	-66.36	89.67	-68.38	103.68	0.9394
	[12m-6m]	-24.35	36.03	-9.06	39.71	0.1444
	[24m-6m]	-27.44	42.61	-30.67	57.34	0.8153
	[24m-12m]	-3.09	31.82	-21.61	35.33	0.0482
Fe	[6m-1m]	15.04	26.87	15.00	30.48	0.9959
	[12m-1m]	24.67	31.49	25.54	35.76	0.9277
	[24m-1m]	8.58	35.63	20.92	37.23	0.2378
	[12m-6m]	9.63	31.39	10.54	35.68	0.9241
	[24m-6m]	-6.46	31.76	5.92	30.39	0.1655
	[24m-12m]	-16.08	27.31	-4.62	39.85	0.2450

[Xmo-Ymo] – change in the parameter value between the time point X months post-operation and the time point Y months post-operation (e.g., [6m-1m] indicates the change between the 6th and 1st month); x̄ – mean; SD – standard deviation; p – level of statistical significance; SG – Sleeve Gastrectomy; RYGB – Roux-en-Y Gastric Bypass; Fe – iron.

In the comparative analysis, no significant differences were found between the groups regarding changes in vitamin B12 and iron (Fe) concentrations in any of the analyzed time intervals ($p > 0.05$). For ferritin, a significant difference between the groups occurred only in the period from 12 to 24 months, where a greater decrease in its level was observed in the SG group compared to the RYGB group ($p = 0.0482$). In the remaining time intervals, the differences did not reach statistical significance.

Table S3. Comparison of changes in body weight and BMI differences in the overall group (women and men) depending on the type of surgery—RYGB and SG—at different time intervals

PARAMETER	DIFFERENCE	RYGB x̄	RYGB SD	SG x̄	SG SD	p
Body weight	[6m-1m]	-17.70	5.65	-18.41	6.20	0.6645
	[12m-1m]	-23.93	7.89	-25.63	9.34	0.4721
	[24m-1m]	-22.48	10.39	-22.85	10.24	0.8956
	[12m-6m]	-6.22	5.60	-7.22	5.56	0.5132
	[24m-6m]	-4.78	8.10	-4.44	6.45	0.8678
	[24m-12m]	1.44	4.25	2.78	2.79	0.1786
BMI	[6m-1m]	-6.29	1.94	-6.57	2.01	0.6080
	[12m-1m]	-8.49	2.69	-9.17	3.16	0.3951
	[24m-1m]	-7.96	3.69	-8.21	3.48	0.8034
	[12m-6m]	-2.20	1.99	-2.60	1.96	0.4514
	[24m-6m]	-1.67	2.95	-1.64	2.26	0.9630
	[24m-12m]	0.53	1.60	0.97	0.97	0.2278

[Xmc-Ymc] – change in the parameter value between the time point X months after surgery and the time point Y months after surgery (e.g., [6m-1m] means the change between the 6th and 1st month); $\bar{x}$ – arithmetic mean; SD – standard deviation; p – level of statistical significance; SG – Sleeve Gastrectomy; RYGB – Roux-en-Y Gastric Bypass; BMI – Body Mass Index.

The analysis of changes in body weight and BMI over the 1 to 24 months following bariatric surgery showed no statistically significant differences between the RYGB and SG groups ($p > 0.05$ for all comparisons). This indicates that both types of surgery demonstrated similar effectiveness in terms of weight loss and BMI reduction during the two-year postoperative period.

Table S4. Comparison of changes in biochemical parameters (AST, ALT, glucose, glycated hemoglobin) in the overall group (women and men) depending on the type of surgery (RYGB vs. SG) at different time intervals

PARAMETER	DIFFERENCE	RYGB x̄	RYGB SD	SG x̄	SG SD	p
AST	[6m-1m]	-7.30	10.11	-7.00	9.34	0.9123
	[12m-1m]	-7.78	8.93	-7.19	8.69	0.8099

PARAMETER	DIFFERENCE	RYGB x̄	RYGB SD	SG x̄	SG SD	p
	[24m-1m]	-8.59	11.90	-5.69	8.81	0.3197
	[12m-6m]	-0.48	6.42	-0.19	4.92	0.8550
	[24m-6m]	-1.30	10.88	1.31	6.55	0.2984
	[24m-12m]	-0.81	7.98	1.50	3.86	0.1873
ALT	[6m-1m]	-13.12	14.29	-13.59	22.22	0.9283
	[12m-1m]	-11.40	15.08	-12.07	19.95	0.8919
	[24m-1m]	-9.60	16.81	-10.44	20.46	0.8721
	[12m-6m]	1.72	8.45	1.52	12.20	0.9455
	[24m-6m]	3.52	11.97	3.15	15.76	0.9245
	[24m-12m]	1.80	9.43	1.63	8.99	0.9471
GLU	[6m-1m]	-1.85	10.95	-3.37	9.37	0.5881
	[12m-1m]	-2.46	13.06	-5.44	8.98	0.3356
	[24m-1m]	-3.50	8.72	-4.37	8.32	0.7115
	[12m-6m]	-0.62	9.33	-2.07	8.06	0.5446
	[24m-6m]	-1.65	10.48	-1.00	7.89	0.7981
	[24m-12m]	-1.04	9.26	1.07	8.32	0.3862
HbA1c (%)	[6m-1m]	-0.01	0.33	-0.02	0.23	0.8024
	[12m-1m]	-0.03	0.43	-0.04	0.27	0.8804
	[24m-1m]	-0.06	0.39	-0.04	0.20	0.8372
	[12m-6m]	-0.02	0.20	-0.02	0.22	0.9364
	[24m-6m]	-0.05	0.30	-0.01	0.15	0.5708
	[24m-12m]	-0.03	0.34	0.01	0.22	0.6756

[Xmc–Ymc] – change in the parameter value between the time point X months after surgery and the time point Y months after surgery (e.g., [6m–1m] indicates the change between the 6th and 1st month); x̄ – arithmetic mean; SD – standard deviation; p – level of statistical significance; SG – Sleeve Gastrectomy; RYGB – Roux-en-Y Gastric Bypass; AST – Aspartate Aminotransferase; ALT – Alanine Aminotransferase; GLU – glucose; HbA1c (%) – glycated hemoglobin.

The analysis of AST, ALT, glucose levels, and glycated hemoglobin from 1 to 24 months after RYGB and SG surgeries showed no statistically significant differences between the groups ($p > 0.05$). Both surgical methods demonstrated a similar impact on liver biochemical and metabolic parameters during long-term follow-up.
